# The long noncoding RNA GAS5 negatively regulates the adipogenic differentiation of MSCs by modulating the miR-18a/CTGF axis as a ceRNA

**DOI:** 10.1038/s41419-018-0627-5

**Published:** 2018-05-10

**Authors:** Ming Li, Zhongyu Xie, Peng Wang, Jinteng Li, Wenjie Liu, Su’an Tang, Zhenhua Liu, Xiaohua Wu, Yanfeng Wu, Huiyong Shen

**Affiliations:** 1 Department of Orthopedics, Sun Yat-sen Memorial Hospital, Sun Yat-sen University, Guangzhou, 510120 People’s Republic of China; 2 Department of Orthopedics, Zhujiang Hospital, Southern Medical University, Guangzhou, 510120 People’s Republic of China; 3 Center for Biotherapy, Sun Yat-sen Memorial Hospital, Sun Yat-sen University, Guangzhou, 510120 People’s Republic of China

## Abstract

Mesenchymal stem cells (MSCs) are important pluripotent stem cells and a major source of adipocytes in the body. However, the mechanism of adipogenic differentiation has not yet been completely elucidated. In this study, the long noncoding RNA GAS5 was found to be negatively correlated with MSC adipogenic differentiation. GAS5 overexpression negatively regulated adipocyte formation, whereas GAS5 knockdown had the opposite effect. Further mechanistic analyses using luciferase reporter assays revealed that GAS5 regulates the adipogenic differentiation of MSCs by acting as competing endogenous RNA (ceRNA) to sponge miR-18a, which promotes adipogenic differentiation. Mutation of the binding sites for GAS5 in miR-18a abolished the effect of the interaction. The miR-18a mimic and inhibitor reversed the negative regulatory effect of GAS5 on MSCs adipogenic differentiation. In addition, GAS5 inhibited miR-18a, which downregulates connective tissue growth factor (CTGF) expression, to negatively regulate the adipogenic differentiation of MSCs. Taken together, the results show that GAS5 serves as a sponge for miR-18a, inhibiting its capability to suppress CTGF protein translation and ultimately decreasing the adipogenic differentiation of MSCs. GAS5 is an important molecule involved in the adipogenic differentiation of MSCs and may contribute to the functional regulation and clinical applications of MSCs.

## Introduction

Mesenchymal stem cells (MSCs) are stem cells with self-renewal and multi-potential differentiation abilities, which allow them to differentiate into osteogenic, adipogenic, and chondrogenic lineages^1^. MSCs have been used in numerous clinical applications due to their advantageous characteristics, including their ability to be easily derived from many sources^2^. For applications involving adipogenic differentiation, MSCs have been used in breast augmentations^3^ and implanted subcutaneously into soft-tissue defects^4^. As the main source of adipocytes, an understanding of the specific mechanism through which MSCs undergo adipogenic differentiation is of great importance. Previous studies have identified some factors that play roles in the adipogenic differentiation of MSCs^5,6^. However, the specific mechanisms have not yet been completely elucidated, and further research is needed.

Noncoding RNAs are a type of non-protein coding RNA that critically regulate processes determining cell fate. Long noncoding RNAs (lncRNAs) are transcripts containing >200 nucleotides of RNA that do not encode proteins. According to some recent studies, lncRNAs affect MSCs differentiation. For instance, HOTAIR impacts MSCs differentiation and is related to senescence-associated DNA methylation^7^. However, the main determinants that influence adipocyte formation by MSCs remain unclear. The lncRNA growth arrest-specific transcript 5 (GAS5) is a non-protein coding RNA that is considered a tumor suppressor gene^8,9^. GAS5 exerts crucial effects on biological proliferation and differentiation^10–12^, but researchers have not determined whether GAS5 affects the adipogenic differentiation of MSCs.

MicroRNAs are another type of noncoding RNA that mainly binds to the 3’ UTR of target mRNAs to inhibit protein translation^13^. MicroRNAs are important regulators of MSCs differentiation^14,15^. In the most recent studies, lncRNAs were shown to act as competing endogenous RNAs (ceRNAs) to sponge miRNAs, thus regulating cell differentiation and other functions^16,17^. For example, GAS5 sponges miR-222 to function as a tumor suppressor in human glioma cells^9^, and MEG3 functions as a ceRNA to regulate ischemic neuronal death by targeting the miR-21/PDCD4 signaling pathway^18^. However, studies confirming that GAS5 acts as ceRNA in MSCs have not yet been reported.

MicroRNA-18a is an important member of the miR-17-92 cluster that plays a carcinogenic role in some tumors^19–21^ and is important for differentiation and apoptosis^22,23^. Connective tissue growth factor (CTGF), a member of a family of cysteine-rich matricellular proteins, recently emerged as a multifunctional regulator because it controls diverse cellular processes, as well as vascular and skeletal development^24–26^. In some studies, the biological function of miR-18a has been reported to be mediated by its binding to the 3′ UTR of CTGF^27^. In addition, some reports of leukemia bone marrow engraftment have indicated that CTGF inhibits the differentiation of MSCs into adipocytes^28^. However, researchers have not clearly determined whether the micro18a/CTGF axis participates in the adipogenic differentiation of MSCs.

Our research illuminates the mechanism through which GAS5 affects the adipogenic differentiation of MSCs. GAS5 negatively regulated adipocyte differentiation in a miR-18a/CTGF-dependent manner, and GAS5 knockdown promoted the differentiation of MSCs into adipocytes. Our findings improve our understanding of the mechanisms of MSCs adipogenic differentiation and may contribute to future molecular therapies using MSCs.

## Methods

### Cell isolation and culture

This study was approved by the ethics committee of Sun Yat-sen Memorial Hospital at Sun Yat-sen University (Guangzhou, People’s Republic of China). Eighteen healthy donors aged 20–30 years were selected for the study. Bone marrow was extracted from the posterior superior iliac spine under sterile conditions. MSCs were purified and isolated using our previously reported methods^29^. MSCs at passage 3 to 5 were used in the experiments. Cells were seeded in 12-well plates and cultured in Dulbecco’s Modified Eagle’s Medium (DMEM; Gibco, New York, USA) containing 10% fetal bovine serum (FBS; Gibco) at 37 °C in a 5% CO_2_ atmosphere. The medium was replaced every 3 days, and MSCs were passaged to 90% confluence.

### Multipotent differentiation potential of MSCs

#### Adipogenic differentiation

For adipogenic induction, MSCs were cultured in adipogenic medium consisting of DMEM supplemented with 10% FBS, 0.5 mM 3-isobutyl-1-methylxanthine (Sigma-Aldrich, St. Louis, USA), 1 mM dexamethasone (Sigma-Aldrich), 10 mg/ml insulin (Sigma-Aldrich), 0.2 mM indomethacin (Sigma-Aldrich), 100 IU/ml penicillin (Sigma-Aldrich), and 100 IU/ml streptomycin (Sigma-Aldrich). The medium was replaced every 3 days. The cells were stained with ORO on day 14.

#### Osteogenic differentiation

MSCs were seeded in 12-well plates and cultured in osteogenic differentiation medium for 0–21 days. This medium was composed of DMEM containing 10% FBS, 0.1 μM dexamethasone, 10 mM β-glycerol phosphate, 50 μM ascorbic acid (Sigma-Aldrich), 100 IU/ml penicillin (Sigma-Aldrich), and 100 IU/ml streptomycin (Sigma-Aldrich). The medium was replaced every 3 days. The cells were stained with Alizarin Red S on day 14.

#### Chondrogenic differentiation

MSCs were grown as high-density pellets (5 × 10^5^ cells) for 3 weeks in specific medium. Serum-free chondrogenic medium containing high-glucose DMEM was supplemented with 1% ITS Premix (Corning Life Sciences, Wisconsin, USA), 50 mg/l ascorbic acid, 1 mM sodium pyruvate (Sigma-Aldrich), 100 nM dexamethasone, and 10 ng/ml recombinant human TGF-β3 (R&D Systems, Minnesota, USA). On day 21, the pellets were prepared for histology and stained with toluidine blue to detect the secretion of sulfated glycosaminoglycans.

### Oil Red O (ORO) staining and quantification

ORO was dissolved in isopropyl alcohol at a concentration of 0.5% as the stock solution. The stock solution was diluted with ultrapure water at a ratio of 3:2 to obtain the working solution. MSCs were fixed with 4% paraformaldehyde and stained with ORO in the working solution for 15 min. After three washes, the stained cells were observed under a microscope and visualized in photomicrographs. The stained cells were destained with isopropyl alcohol. A 200-µl aliquot was transferred to a 96-well plate, and the absorbance at 520 nm was measured.

### Flow cytometry

MSCs were digested with 0.25% trypsin supplemented with 0.53 mM EDTA (Gibco). After centrifugation, MSCs were resuspended in phosphate-buffered saline (PBS) and incubated for 30 min with antibodies against human CD29-phycoerythrin (PE), CD34-allophycocyanin (APC), CD44-fluorescein isothiocyanate (FITC), CD45-FITC, CD105-FITC, or HLA-DR-PE. All of the antibodies used in flow cytometry are purchased from BD Biosciences (New York, USA). Flow cytometry was performed to identify the MSC phenotypes. For the cell cycle analysis, MSCs were trypsinized and fixed with 80% cold alcohol at 4 °C for 12 h. After centrifugation, MSCs were resuspended in 50 µl of RNase and 450 µl of propidium iodide (PI; Sigma-Aldrich) staining fluid, and the phases of the cell cycle in each sample after 30 min of incubation were analyzed by flow cytometry.

### Lentivirus construction and infection

The lncRNA GAS5 and CTGF siRNA were designed and synthesized by GenePharma (Shanghai, China). We selected one siRNA (Supplementary Table 1) with the best knockdown efficiency to build the short hairpin RNA (shRNA) and construct the lentivirus. The lncRNA GAS5 wild type (WT) and lncRNA GAS5 mutant type (MUT) overexpression lentiviruses were generated by co-transfecting pGLVH1/GFP/Puro (Gene Pharma) and packing plasmids (pGag/Pol, pRev, and pVSV-G) into 293T cells. Culture supernatants containing lentiviruses were filtered and concentrated 72 h after transfection. Each lentivirus (10^9^ transducing units/ml) and polybrene (5 mg/ml) were added to the medium, and MSCs were incubating with the resulting mixture for 24 h at a multiplicity of infection of 50. In both the overexpression and downregulation experiments, we infected MSCs with the lentivirus on day 0, removed the lentivirus-containing medium after 24 h, and then began to induce adipogenic differentiation. Related experiments were performed using induced MSCs.

### Real-time quantitative reverse transcription-polymerase chain reaction (qRT-PCR)

MSCs were cultured in 12-well plates. TRIzol (Invitrogen, Massachusetts, USA) was added to the wells at the appropriate times, and total RNA was isolated from the MSCs and transcribed into complementary DNAs using a PrimeScript RT reagent kit (TaKaRa, Dalian, China). qRT-PCR was then performed with a LightCycler 480 PCR system (Roche, Basel, Switzerland) using SYBR Premix Ex Taq (TaKaRa). The detailed method was described in our previously published studies^30^. The results were first normalized to the level of GAPDH expression. The data were standardized based on GAPDH expression, and the 2^−ΔΔCt^ method was used to analyze the data and determine the relative expression of each gene. The primers for miRNA were provided by Guangzhou Ribobio Biotechnology Co., Ltd. The miRNA RT reaction was based on primers with a stem loop structure. The reverse transcription primers for the stem loop structure bind to the 3′-end of the miRNA, and the reverse transcription reaction was performed using reverse transcriptase. Specific forward primers, universal reverse primers, and SYBR Green fluorescent dyes for quantitative amplification were used to enable the real-time quantitative detection of reverse transcription products. The forward and reverse primers for each gene are listed in Supplementary Table 2.

### Western blot and agarose gel electrophoresis

MSCs were lysed at the appropriate times and centrifuged at 14,000 rpm/min, and the proteins were then boiled with loading buffer. Equal amounts of protein extracts were separated on 10 or 12% gels and transferred to polyvinylidene fluoride (PVDF) membranes (Millipore, Massachusetts, USA). The membranes were blocked with BSA and incubated with primary antibodies against AGO2 (Abcam, Cambridge, United Kingdom), PPAR-γ (Cell Signaling Technology, Massachusetts, USA), C/EBP-α (Abcam), FABP4 (Abcam), CTGF (R&D Systems) and GAPDH (Beyotime, Shanghai, China) overnight at 4 °C. After three washes, the PVDF membranes were incubated with a horseradish peroxidase (HRP)-conjugated secondary antibody (diluted 1:3000; Santa Cruz Biotechnology), and specific antibody–antigen complexes were detected using Immobilon Western Chemiluminescent HRP Substrate (Millipore).

PCR products were collected in microtubes with DNA loading buffer and separated on a 1.2% agarose containing 0.01% Gel red (Biotium). DNA products were detected by UV light and photographed for observation.

### Isolation of cytoplasmic and nuclear fractions from cells

Cytoplasmic and nuclear RNA was extracted from MSCs using the PARIS™ Kit (Thermo Scientific), according to the manufacturer’s instructions. The RNAs extracted from each of the fractions were analyzed by qRT-PCR. The data were analyzed to determine the nuclear and cytoplasmic levels of each RNA.

### Luciferase reporter assay

The lncRNA GAS5 WT and lncRNA GAS5 MUT sequences were synthesized and cloned into pmirGLO plasmid vectors, and the constructed vectors were transfected into MSCs with Lipofectamine 3000 Transfection Reagent (Invitrogen). The miRNA mimic or negative control was added to specific wells of the plate. Relative luciferase activities were measured using the Dual-Luciferase® Reporter (DLR™) Assay System (Promega, Wisconsin, USA) according to the manufacturer’s instructions and analyzed as the ratio of firefly luciferase activity to Renilla luciferase activity.

### RNA-binding protein immunoprecipitation

The Magna RIP™ RNA-Binding Protein Immunoprecipitation Kit (Millipore) was used for the RIP assay. Briefly, about 5 × 10^6^ of MSCs was lysed with RIP lysis buffer and then incubated with RIP buffer containing magnetic beads conjugated with the anti-AGO2 antibody or negative control purified rabbit IgG. A positive control anti-SNRNP70 antibody was used for the RIP procedure. Samples were incubated with proteinase K, and immunoprecipitated RNAs were isolated. The precipitated RNAs were purified and subjected to quantitative PCR to detect the presence of the target lncRNA or miRNA.

### RNA pull-down assay

A Pierce™ Magnetic RNA-Protein Pull-Down Kit (Thermo Fisher Scientific, Massachusetts, USA) was used for the RNA pull-down assay. Biotin-labeled RNAs were transcribed in vitro with the TranscriptAid T7 High-Yield Transcription Kit (Thermo Fisher Scientific) and Pierce RNA 3′ End Desthiobiotinylation Kit (Thermo Fisher Scientific) and then treated with RNase-free DNase I (Thermo Scientific). The retrieved proteins were detected by Western blotting. The procedure was conducted according to the manufacturer’s instructions and standard protocols of Western blot described previously.

### Statistical analyses

The experiments and analyses were separately completed by the different authors listed in this study. Statistical analyses were performed using SPSS 22.0 software (Chicago, IL, USA). The data are expressed as the means ± standard deviations (SD). Differences between two groups were assessed using Student’s *t*-test, and differences among three or more groups were analyzed by ANOVA. *P* values < 0.05 were considered significant.

## Results

### The lncRNA GAS5 negatively correlates with the adipogenic differentiation capacity of MSCs

We characterized the markers of MSCs by flow cytometry and found that the cells in our experiment expressed CD29, CD105, and CD44 but lacked CD45, CD34, and HLA-DR (Supplementary Figure 1A). Alizarin Red S, ORO and toluidine blue staining was performed to detect differentiated cells. The results are shown in Supplementary Figure 1B.

**Fig. 1:**
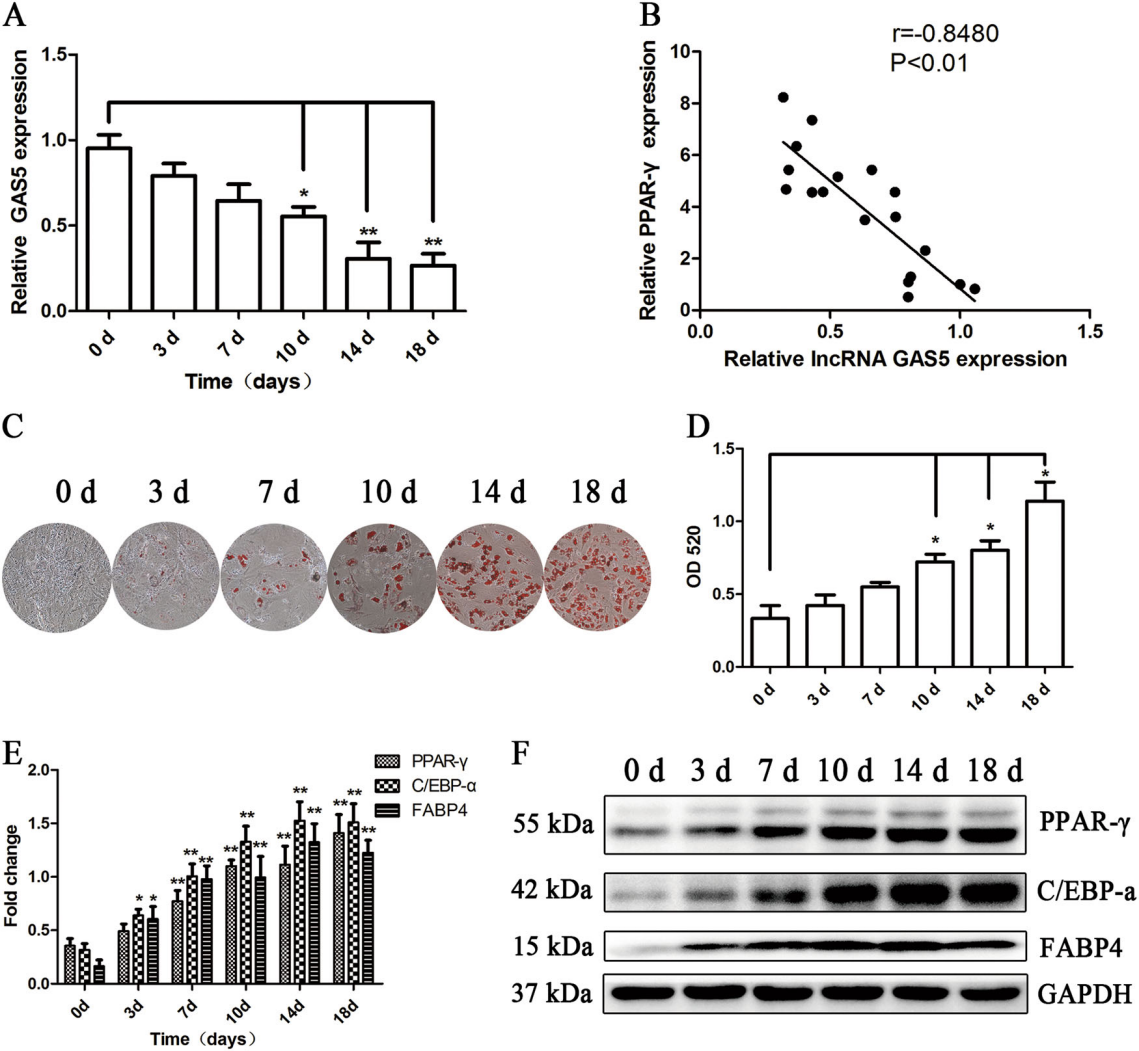
The lncRNA GAS5 negatively correlates with the adipogenic differentiation capacity of MSCs. **a** Relative expression of GAS5 during the adipogenic differentiation of MSCs. **b** Relative expression levels of GAS5 and PPAR-γ during the adipogenic differentiation of MSCs obtained from 18 healthy donors. **c**, **d** ORO staining and quantification on days 0, 3, 7, 10, 14, and 18 of MSC adipogenic differentiation. **e**, **f** Relative levels of PPAR-γ, C/EBP-α and FABP4 mRNAs and proteins on days 0, 3, 7, 10, 14, and 18 of MSC adipogenic differentiation. The results are presented as the means ± SD (*P < 0.05, **P < 0.01, as determined by Student’s *t*-test). All experiments were performed three independent times

We evaluated the pattern of GAS5 expression by qRT-PCR to investigate the impact of this lncRNA on the adipogenic differentiation of MSCs and found that GAS5 expression was downregulated during the adipogenic differentiation of MSCs by approximately 70% on day 14 (Fig. 1a). We analyzed the relative expression of GAS5 and PPAR-γ and observed a negative correlation during the differentiation of MSCs (Fig. 1b), suggesting that GAS5 may exert a negative effect on the differentiation of MSCs into adipocytes. The adipogenic differentiation of MSCs was confirmed by ORO staining, Western blotting and qRT-PCR (Fig. 1c-f).

### The lncRNA GAS5 negatively regulates the adipogenic differentiation of MSCs

**Fig. 2:**
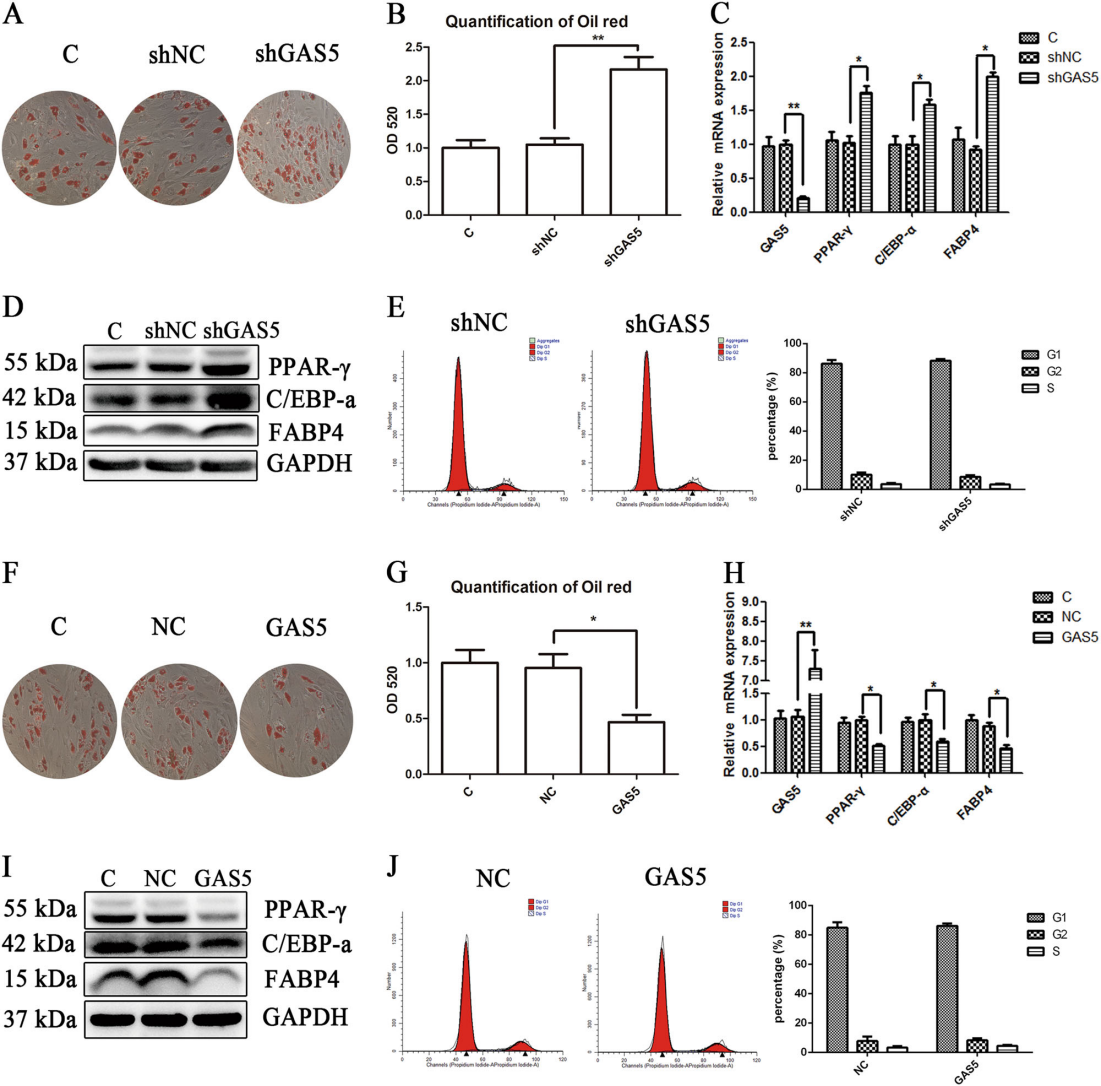
The lncRNA GAS5 negatively regulates the adipogenic differentiation of MSCs. **a**, **b** ORO and quantification in GAS5-overexpressing cells. **c** Expression of mRNAs encoding the adipogenic markers PPAR-γ, C/EBP-α, and FABP4 in MSCs overexpressing GAS5. **d** Protein levels of the adipogenic markers PPAR-γ, C/EBP-α, and FABP4 in MSCs overexpressing GAS5. **e** Cell cycle analysis of MSCs overexpressing GAS5. **f**, **g** ORO staining and quantification in GAS5-knockdown cells. **h** Expression of mRNAs encoding the adipogenic markers PPAR-γ, C/EBP-α, and FABP4 in GAS5-knockdown MSCs. **i** Protein levels of the adipogenic markers PPAR-γ, C/EBP-α, and FABP4 in GAS5-knockdown MSCs. **j** Cell cycle analysis of GAS5-knockdown MSCs through PI staining. The results are presented as the means ± SD (*P < 0.05, **P < 0.01, as determined by Student’s *t*-test, *n* = 3). All experiments were performed three independent times

We constructed a GAS5-overexpressing lentivirus and infected MSCs to investigate whether the lncRNA GAS5 affects the adipogenic differentiation of MSCs. MSCs were induced toward adipogenic differentiation for 14 days, and ORO staining was performed to determine the effect of GAS5. GAS5 overexpression significantly decreased the formation of adipocytes, according to the ORO staining and quantitative analysis (Fig. 2a, b). The mRNA expression and protein levels of adipogenic markers were decreased in the GAS5 overexpression group (Fig. 2c,d). We subsequently selected the most efficient siRNA among three siRNAs and constructed lentiviruses carrying the shRNA (Supplementary Figure 2A). GAS5 knockdown using the shRNA significantly increased the number of adipogenic of MSCs (Fig. 2f, g) and increased the mRNA and protein expression of PPAR-γ, C/EBP-α and FABP4 (Fig. 2h, i). We also explored whether GAS5 affected MSC proliferation. The cell cycle of MSCs was not influenced by either GAS5 overexpression or knockdown, as demonstrated by PI staining and the flow cytometry analysis (Fig. 2e, j). Thus, GAS5 negatively regulated the adipogenic differentiation of MSCs.

### The lncRNA GAS5 sponges miRNAs by acting as a ceRNA

**Fig. 3:**
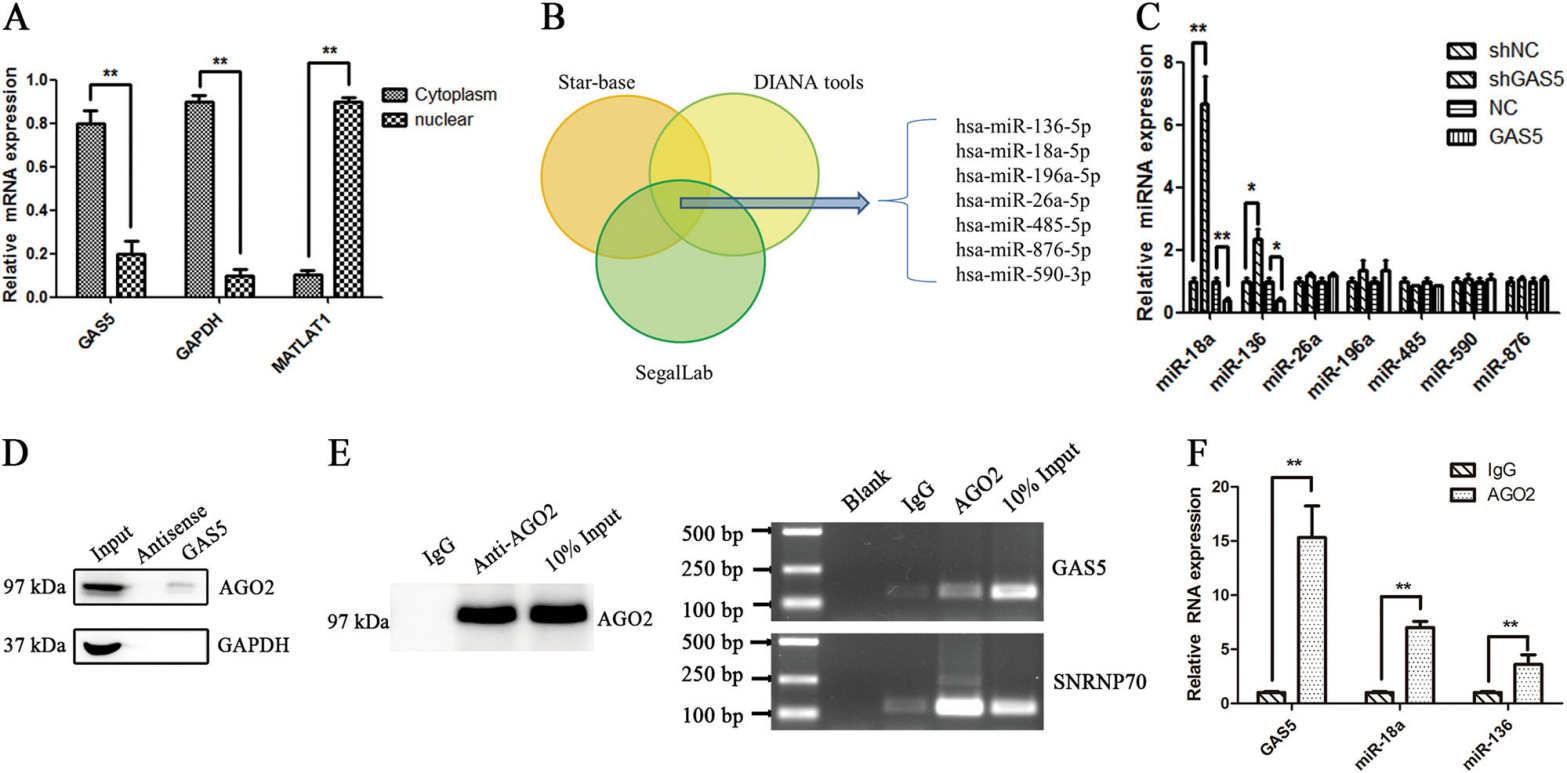
The lncRNA GAS5 sponges miRNAs as a competing endogenous RNA. **a** Analysis of GAS5, GAPDH, and MALAT1 levels in the cytoplasmic and nuclear fractions of MSCs. **b** Bioinformatics analysis of three databases (ChipBase, LncRNAdb, and StarBase). **c** Levels of different miRNAs in GAS5-knockdown and GAS5-overexpressing MSCs, as analyzed by qPCR. **d** Results of an RNA pull-down assay analyzing the interaction between GAS5 and AGO2. **e** Results of the RIP assay analyzing the efficiency (left panel) and RT-PCR products of GAS5 and SNRNP70 (right panel). SNRNP70 was used as a positive control in the RIP assay and the immunoprecipitation efficiency analysis. **f** Levels of GAS5, miR-18a and miR-136 in the IgG group and the anti-AGO2 group. The results are presented as the means ± SD (*P < 0.05, **P < 0.01, as determined by Student’s *t*-test, *n* = 3). All experiments were performed three independent times

**Fig. 4:**
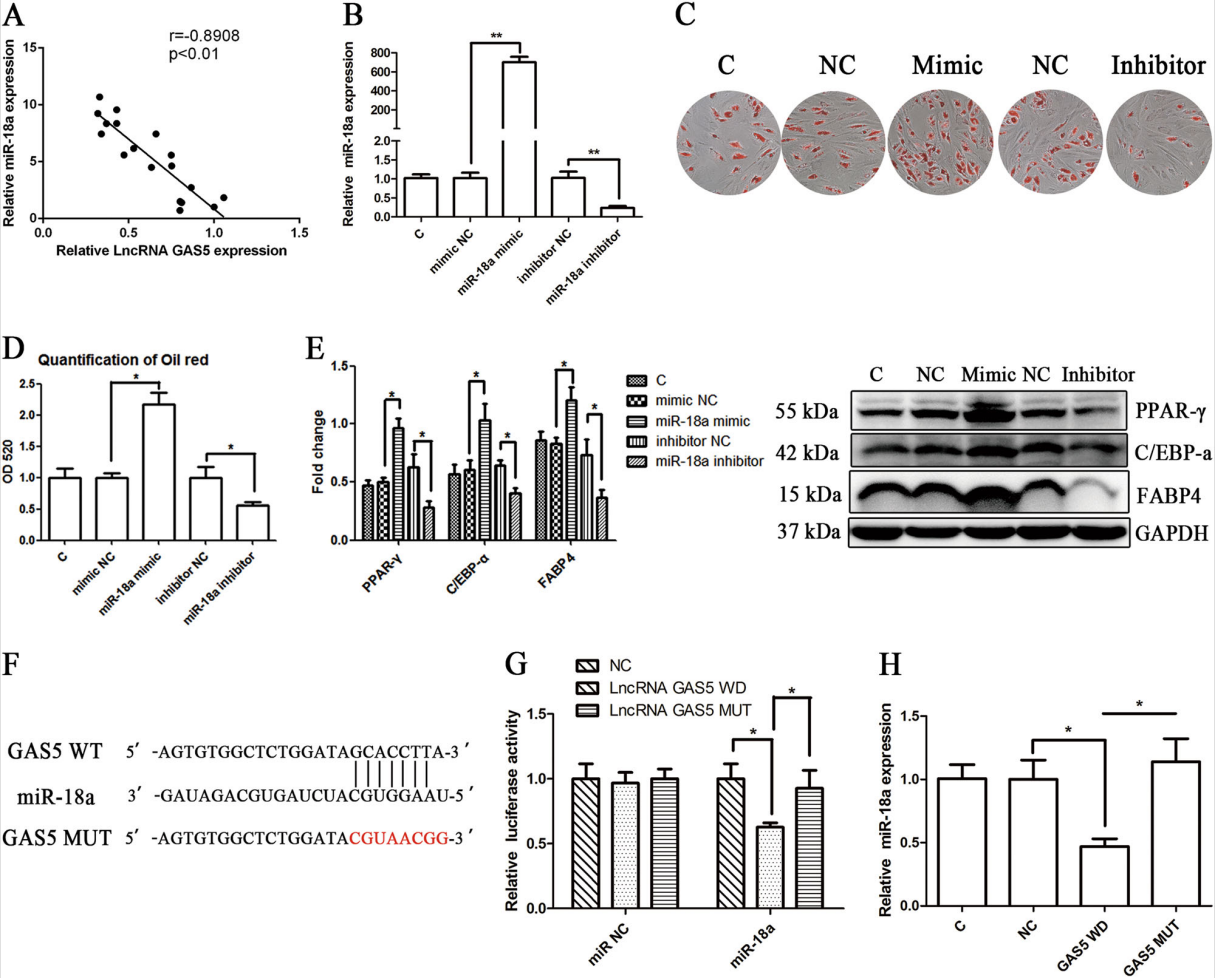
GAS5 specifically binds to miRNA-18a to negatively regulate the adipogenic differentiation of MSCs. **a** Relative expression levels of GAS5 and miR-18a during the adipogenic differentiation of MSCs obtained from 18 healthy donors. **b** Relative levels of miR-18a expression in cells transfected with the miR-18a mimic or inhibitor. **c**, **d** ORO staining and quantification in cells transfected with the miR-18a mimic or inhibitor. **e** Effects of the miR-18a mimic or inhibitor on the relative levels of PPAR-γ, C/EBP-α, and FABP4 proteins. **f** The binding sites of WT GAS5 and mutated sites in MUT GAS5 are shown. **g** Relative luciferase activities of WT and MUT GAS5 in cells transfected with the miR-18a mimic. **h** Levels of miR-18a in cells overexpressing GAS5 WT or MUT. The results are presented as the means ± SD (*P < 0.05, **P < 0.01, as determined by Student’s *t*-test, *n* = 3). All experiments were performed three experiments times

We isolated cytoplasmic and nuclear fractions from MSCs to identify the specific mechanism through which GAS5 regulates MSC adipogenic differentiation and found that GAS5 was mainly located in the cytoplasm (Fig. 3a). Cytoplasmic lncRNAs have been shown to act as ceRNAs that sponge miRNAs^16^. We performed a bioinformatics analysis of three databases (ChipBase, LncRNAdb, and StarBase) to identify miRNAs that potentially interact with GAS5. The results revealed seven miRNAs with the highest scores for binding to GAS5 (Fig. 3b). We designed qPCR primers for these seven miRNAs to determine whether they were regulated by GAS5. Notably, the levels of miR-18a and miR-136 were significantly increased in the GAS5 knockdown group and decreased in the GAS5 overexpression group (Fig. 3c). We used an RNA pull-down assay to examine whether GAS5 bound to AGO2, the core protein in the RNA-induced silencing complex^31^ (RISC). According to the Western blot results, GAS5, but not the antisense control, bound to AGO2 (Fig. 3d). An RIP assay was performed to further verify the interaction between GAS5 and miR-18a and miR-136. SNRNP70 was used as a positive control in the RIP assay, and the immunoprecipitation efficiency is presented in Fig. 4e. The levels of GAS5, miR-18a, and miR-136 were markedly increased in the anti-AGO2 group compared with the IgG group (Fig. 3f).

### GAS5 specifically binds to miRNA-18a to negatively regulate the adipogenic differentiation of MSCs

We subsequently analyzed the relationship between miR-18a, miR-136 and GAS5, and the results revealed negative correlations for miR-18a (Fig. 4a) and miR-136 (Supplementary Figure 2F) with GAS5. We investigated whether miR-18a influenced the adipogenic differentiation of MSCs. We transfected MSCs with a miR-18a mimic and inhibitor (Fig. 4b), and the resulting adipogenic changes were detected by ORO staining. The miR-18a mimic significantly increased the adipogenic differentiation of MSCs (Fig. 4c, d), and this effect was accompanied by changes in the expression of adipogenic markers (Fig. 4e). Based on these results, miR-18a functions in the adipogenic differentiation of MSCs. We also determined whether miR-136 influenced the adipogenic differentiation of MSCs. However, ORO staining and quantification yielded negative results, suggesting that miR-136 is not involved in MSC adipogenic differentiation (Supplementary Figure 2G).

We also checked the possible binding sites for GAS5 in miR-18a and constructed WT and mutation type (MUT) luciferase reporter genes including firefly and Renilla luciferase sequences (Fig. 4f). According to the results of the luciferase assay, the miR-18a mimic significantly decreased the fluorescence of GAS5 WT but had no impact on GAS5 MUT (Fig. 4g). The overexpression of GAS5 WT significantly downregulated miR-18a expression, but GAS5 MUT overexpression in MSCs had no influence on the miR-18a levels (Fig. 4h). Changes in miR-18a expression had no influence on GAS5 expression (Supplementary Figure 2B). Thus, GAS5 serves as a sponge of miR-18a, and miR-18a might be the downstream molecular target of GAS5.

**Fig. 5:**
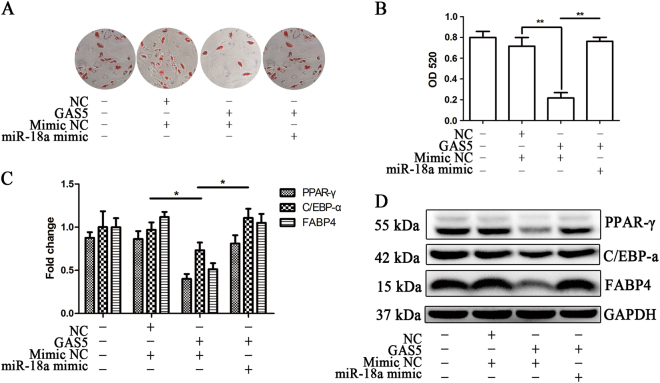
The miR-18 mimic reverses the effect of GAS5 overexpression on the adipogenic differentiation of MSCs. **a** ORO staining in GAS5-overexpressing MSCs transfected with the miR-18a mimic. **b** Quantification of ORO staining in GAS5-overexpressing MSCs transfected with the miR-18a mimic. **c** Quantification of the relative levels of PPAR-γ, C/EBP-α, and FABP4 proteins in GAS5-overexpressing MSCs transfected with the miR-18a mimic. **d** Western blots showing the PPAR-γ, C/EBP-α, and FABP4 levels in GAS5-overexpressing MSCs transfected with the miR-18a mimic. The results are presented as the means ± SD (*P < 0.05, **P < 0.01, as determined by Student’s *t*-test, *n* = 3). All experiments were performed three independent times

**Fig. 6:**
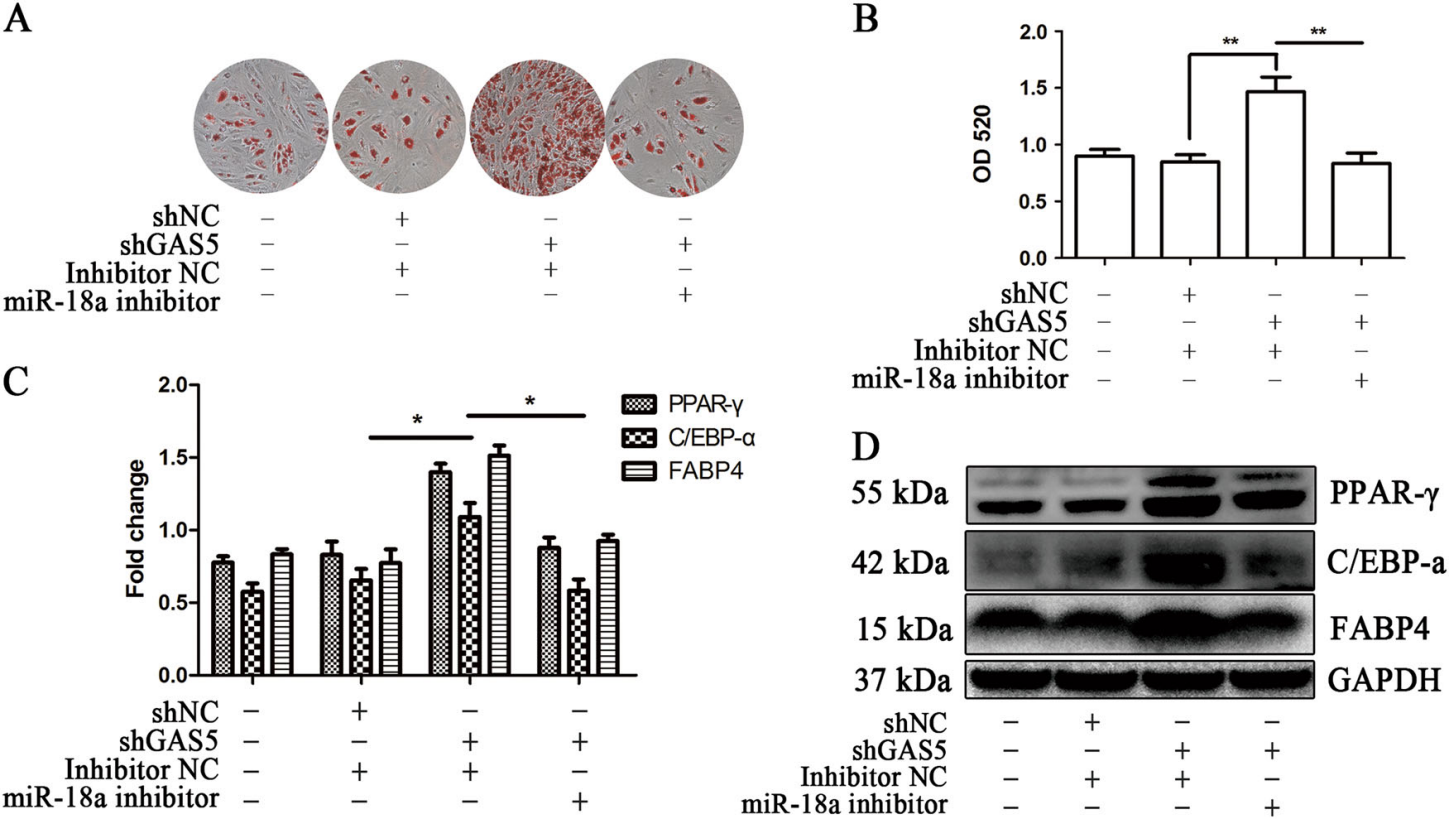
The miR-18a inhibitor reverses the effect of GAS5 knockdown on the adipogenic differentiation of MSCs. **a** ORO staining in GAS5-knockdown MSCs transfected with the miR-18a inhibitor. **b** Quantification of ORO staining in GAS5-knockdown MSCs transfected with the miR-18a inhibitor. **c** Quantification of the relative levels of PPAR-γ, C/EBP-α, and FABP4 proteins in GAS5-knockdown MSCs transfected with the miR-18a inhibitor. **d** Western blots showing the PPAR-γ, C/EBP-α, and FABP4 levels in GAS5-knockdown MSCs transfected with the miR-18a inhibitor. The results are presented as the means ± SD (*P < 0.05, **P < 0.01, as determined by Student’s *t*-test, *n* = 3). All experiments were performed three independent times

We then explored whether miR-18a reversed the effect of GAS5. We co-transfected MSCs with the miR-18a mimic and the GAS5-overexpressing lentivirus and induced adipogenic differentiation for 14 days. ORO staining showed that the miR-18a mimic significantly reversed the effect of GAS5 on decreasing MSC adipogenic differentiation (Fig. 5a, b). Changes in the protein levels of adipogenic markers also supported this reversed effected (Fig. 5c, d). We subsequently explored the effect of the miR-18a inhibitor on GAS5-silenced MSCs. The ORO staining results and the analysis of the PPAR-γ, C/EBP-α, and FABP4 protein levels showed that the miR-18a inhibitor also reversed the effect of GAS5 knockdown (Fig. 6a-d). In summary, GAS5 inhibits the adipogenic differentiation of MSCs by decreasing the miR-18a levels.

### The miR-18a/CTGF axis affects the adipogenic differentiation of MSCs and is modulated by GAS5

**Fig. 7:**
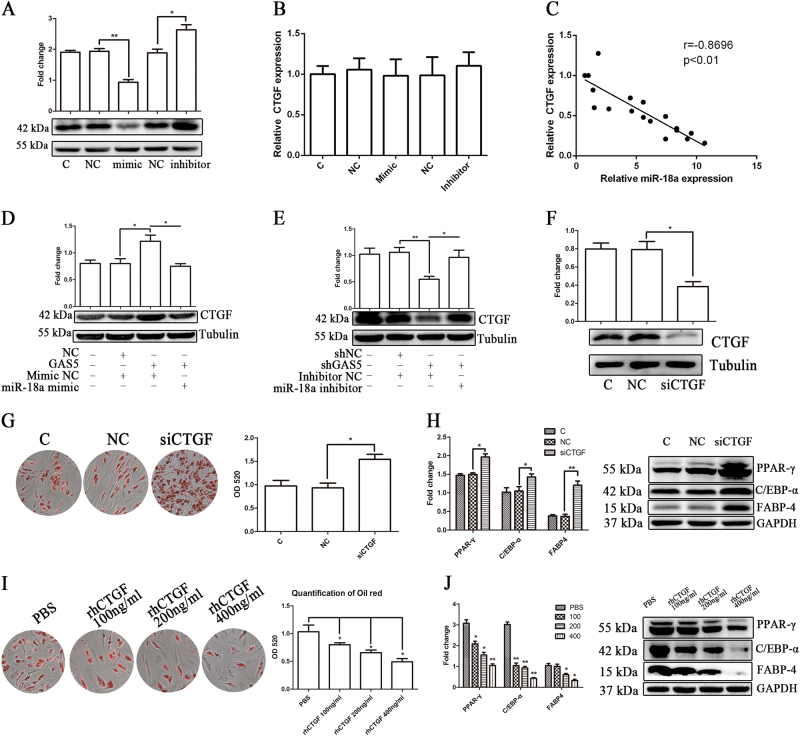
The miR-18a/CTGF axis affects the adipogenic differentiation of MSCs and is modulated by GAS5. **a** Levels of CTGF protein in MSCs transfected with the miR-18a mimic or inhibitor. **b** Levels of CTGF mRNA in MSCs transfected with the miR-18a mimic or inhibitor. **c** Relative expression levels of CTGF and miR-18a during the adipogenic differentiation of MSCs obtained from 18 healthy donors. **d** Levels of CTGF protein in GAS5-overexpressing MSCs transfected with or without the miR-18a mimic. **e** Levels of CTGF protein in GAS5-knockdown MSCs transfected or not transfected with the miR-18a inhibitor. **f** Levels of CTGF protein in MSCs transfected with the CTGF siRNA. **g** ORO staining and quantification in MSCs transfected with the CTGF siRNA. **h** Levels of the adipogenic markers PPAR-γ, C/EBP-α, and FABP4 in MSCs transfected with the CTGF siRNA. **i** ORO staining and quantification in MSCs treated with recombinant human CTGF. **j** Levels of the adipogenic markers PPAR-γ, C/EBP-α, and FABP4 in MSCs treated with recombinant human CTGF. The results are presented as the means ± SD (*P < 0.05, **P < 0.01, as determined by Student’s *t*-test, *n* = 3). All experiments were performed three independent times

Based on bioinformatics predictions and previous studies, miR-18a functions by binding to the CTGF mRNA. In addition, the levels of the CTGF protein were decreased by the expression of the miR-18a mimic and increased by the expression of the miR-18a inhibitor (Fig. 7a). We examined the level of the CTGF mRNA in MSCs transfected with the miR-18a mimic or inhibitor and obtained negative results (Fig. 7b). The relationship between miR-18a and CTGF is presented in Fig. 7c, which reveals a negative correlation. We also detected the level of the CTGF protein in MSCs transfected by GAS5-knockdown or GAS5-overexpression lentiviruses. The level of the CTGF protein was increased by GAS5 overexpression and decreased by shGAS5. The miR-18a mimic or inhibitor reversed the effects of GAS5 overexpression or knockdown, respectively (Fig. 7d, e), indicating that GAS5 affects the adipogenic differentiation of MSCs through a miR-18a/CTGF-dependent mechanism.

We designed three siRNAs to knock down CTGF and determine the effect of CTGF on the adipogenic differentiation of MSCs (Supplementary Figure 2C). We selected siRNA3 (Fig. 7f) to examine whether CTGF knockdown influenced MSC adipogenic differentiation. The CTGF siRNA significantly increased the adipogenic differentiation of MSCs (Fig. 7g, h), and recombinant human CTGF (rhCTGF) decreased the adipogenic differentiation of MSCs in a concentration-dependent manner (Fig. 7i, j). The interaction between GAS5, miR-18a, and CTGF in MSC adipogenic differentiation is shown in Supplement Fig. 3.

## Discussion

In our study, GAS5 was negatively correlated with the adipogenic differentiation capacity of MSCs. Alterations in GAS5 expression were used to regulate the MSC adipogenic differentiation capability. Based on the results of mechanistic studies, GAS5 acts as a ceRNA that sponges miR-18a. Furthermore, the miR-18a/CTGF axis regulates MSC adipogenic differentiation. Specifically, GAS5 negatively regulates MSC adipogenic differentiation by modulating the miR-18a/CTGF axis. Therefore, the lncRNA GAS5 negatively regulates the adipogenic differentiation of MSCs by modulating the miR-18a/CTGF axis as a ceRNA.

lncRNAs have been reported to play important roles in many biological functions. For instance, the lncRNA EGFR-AS1 mediates epidermal growth factor receptor addiction and modulates treatment responses in squamous cell carcinoma^32^. DDX5 and its associated lncRNA Rmrp modulate TH17 cell effector functions^33^. lnc-DC binds to STAT3 to influence dendritic cell differentiation^34^. The lncRNA GAS5 is considered a cancer suppressor^9^ but was recently shown to be a vital regulator of the self-renewal of human embryonic stem cells^35^. Thus, GAS5 may play an essential role in cell development and differentiation. In the present study, GAS5 was negatively correlated with adipogenic differentiation. The upregulation of GAS5 expression resulted in a corresponding change in adipogenic differentiation, suggesting that GAS5 negatively regulates MSC adipogenic differentiation. GAS5 correlates with tumor growth and invasion^8,11^, and in our previous studies, we found some lncRNAs were associated with abnormal pathological osteoblasts in ankylosing spondylitis^36^. Therefore, abnormal expression of the lncRNA GAS5 in MSCs may be linked to various MSC-related diseases.

According to their location in the cell, lncRNAs are divided into two types: nuclear lncRNAs and cytoplasmic lncRNAs. lncRNAs located in the nucleus function by affecting transcription factors or by inducing epigenetic modifications^37,38^, whereas lncRNAs located in the cytoplasm are predominantly competitive endogenous RNAs^18,39^. In the present study, GAS5 was found to be located in the cytoplasm of MSCs, consistent with its location in other cell types^18,39^. GAS5 has been shown to function as a ceRNA, but the mechanism of GAS5 in MSCs has not been reported. Our experiments suggest that GAS5 functions as a ceRNA, consistent with other reports in the literature^40,41^, and affects the adipogenic differentiation of MSCs via the miR-18a/CTGF axis. Recently, some researchers proposed the mechanism of target RNA-directed miRNA degradation (TDMD)^42^ and showed that miRNA degradation is more active in primary neurons. Additionally, TDMD sites appear to be two independent processes, and their balance is regulated by alterations in miRNA abundance. However, the canonical miRNA binding sites neither induce miRNA decay nor compromise TDMD. In our research, GAS5 sponged miR-18a by binding to the canonical binding sites, and the sponge mechanism has been verified in several cell types. This information improves our knowledge of the mechanisms underlying the interactions between lncRNAs and miRNAs. Nevertheless, the sponge mechanism and TDMD appear to be cell-specific processes and deserve further exploration.

MicroRNAs are a class of noncoding single-stranded RNA molecules with a length of approximately 22 nucleotides and encoded by endogenous genes. These molecules are involved in the regulation of post-transcriptional gene expression in cell biology. MicroRNAs, such as miR-140^43^, miR-27a^14^, and miR-223^15^, influence the adipogenic differentiation of MSCs. MicroRNA-18a is an important member of the miR-17-92 cluster, but the role of miR-18a in the differentiation of MSCs has not been evaluated. As shown in the present study, miR-18a promotes the adipogenic differentiation of MSCs. CTGF (CCN2) is a member of the CCN (CYR61, CTGF, and NOV) family of proteins. The effect of miR-18a on CTGF was confirmed in previous studies examining human corneal epithelial cells and in pneumoconiosis^27,44^. In addition, CTGF promotes the proliferation and invasion of a variety of tumor cells^45^. A study of CTGF and MSCs by Battula and coworkers found that CTGF is required for bone marrow fat formation after leukemia bone marrow engraftment^28^. In our study, CTGF negatively regulated the adipogenic differentiation of MSCs, whereas CTGF itself was regulated by miR-18a. Therefore, the miR-18a/CTGF axis plays a vital role in MSC differentiation. As mentioned above, GAS5 functions as a ceRNA, and the experimental results showed that GAS5 negatively regulates the miRNA18/CTGF axis. Thus, GAS5 negatively regulates the adipogenic differentiation of MSCs by modulating the miR-18a/CTGF axis. CTGF expressed in MSCs is an important factor in leukemia and tongue squamous cell carcinoma^28,45^. However, research regarding the functions of GAS5 and miR-18a in these diseases is lacking. Our findings may provide insights into the mechanisms through which GAS5 and miR-18a correlate with CTGF in diseases. However, further research is needed to test the specific role of GAS5 in these diseases.

Adipose tissue comprises a substantial amount of biologically active tissues; non-obese men and women have ~12 kg and ~14 kg of adipose tissue, respectively^46^. Adipocytes are the basic components of adipose tissue, and MSCs are a main source of adipocytes in many tissues. Normal MSC adipogenic differentiation helps maintain the physiological balance in the body. However, the abnormal adipogenic differentiation of MSCs is associated with many disorders, such as cardiovascular disease^47^, poor glycemic control^48^ and hematopoietic diseases^49^. Thus, an understanding of the mechanisms regulating the adipogenic differentiation of MSCs is of great importance. Based on our findings, the lncRNA GAS5 negatively regulates the adipogenic differentiation of MSCs by modulating the miR-18a/CTGF axis as a ceRNA. This information provides a new theoretical basis for clarifying the mechanism of MSC differentiation and provides a novel therapeutic target for the treatment of adipocyte-related diseases. The adipogenic differentiation of MSCs is currently widely applied for augmentation and reconstruction in aesthetic and reconstructive surgery^4,50,51^. The results of our study will be helpful for expanding the applications of MSCs in the surgical reconstruction field. However, our research has some limitations, such as the lack of an animal model to study the function of GAS5 in vivo. In addition, researchers have not yet determined whether GAS5 affects osteogenic or chondrogenic differentiation, and the specific mechanism through which GAS5 regulates the miR-18a/CTGF axis has not been elucidated. Additional studies are needed to surmount these shortcomings. Nonetheless, GAS5 may play a greater role in the function and application of MSCs in the future.

### Electronic supplementary material

Supplement Table

Supplement Figure 1

Supplement Figure 2

Supplement Figure 3
