# Correction: The long noncoding RNA GAS5 negatively regulates the adipogenic differentiation of MSCs by modulating the miR-18a/CTGF axis as a ceRNA

**DOI:** 10.1038/s41419-024-06612-x

**Published:** 2024-05-31

**Authors:** Ming Li, Zhongyu Xie, Peng Wang, Jinteng Li, Wenjie Liu, Su’an Tang, Zhenhua Liu, Xiaohua Wu, Yanfeng Wu, Huiyong Shen

**Affiliations:** 1 Department of Orthopedics, Sun Yat-sen Memorial Hospital, Sun Yat-sen University, Guangzhou, 510120 People’s Republic of China; 2 Department of Orthopedics, Zhujiang Hospital, Southern Medical University, Guangzhou, 510120 People’s Republic of China; 3 Center for Biotherapy, Sun Yat-sen Memorial Hospital, Sun Yat-sen University, Guangzhou, 510120 People’s Republic of China

Correction to: *Cell Death and Disease* 10.1038/s41419-018-0627-5, published online 10 May 2018

In Figure 5A, the authors have checked the original raw data and records of the experiments. Here, they pulled the same picture into the typesetting software twice in the process of typesetting in Figure 5A, resulting in the picture repeated twice in this figure. The authors would like to apologize for any inconvenience caused. They have made correction to Figure 5A and attached the original pictures here.
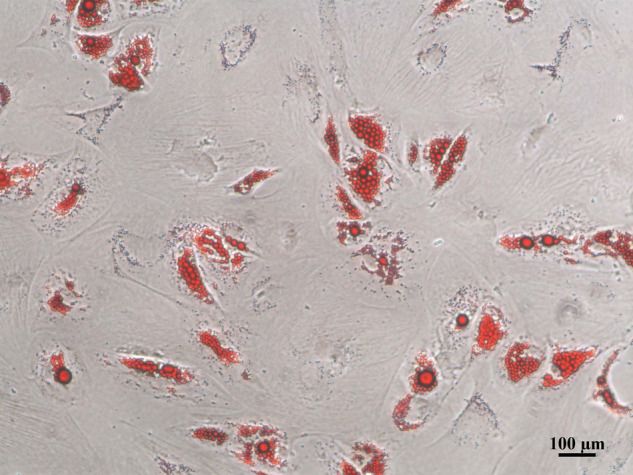

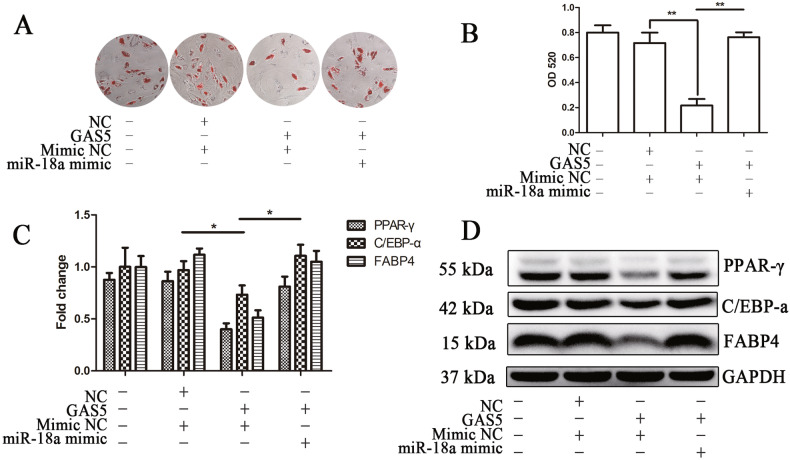


In Figure 7H, it was due to the fact that the original image was not correctly placed in the arrangement process, resulting in the mistake of the PPAR-γ bands in Figure 7H. The authors would like to apologize for any inconvenience caused. There is no impact on the final conclusions. The original pictures of the bands involved are attached here.
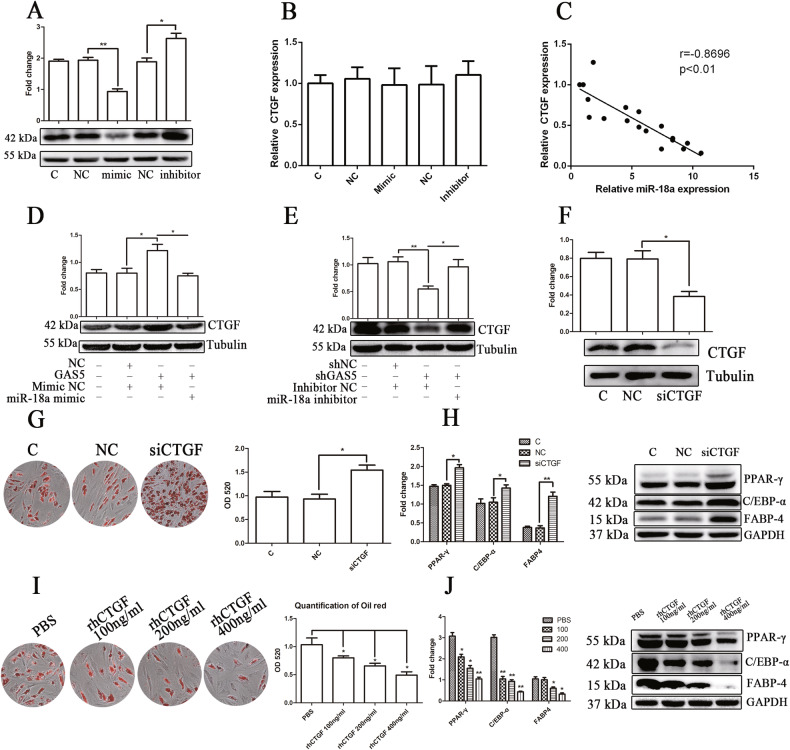

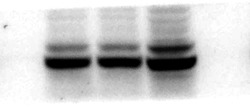


The original article has been corrected.

