# Supplementary material for: The long noncoding RNA GAS5 negatively regulates the adipogenic differentiation of MSCs by modulating the miR-18a/CTGF axis as a ceRNA
Source: Cell Death Dis. 2018 May 10;9(5):554. doi: 10.1038/s41419-018-0627-5 (PMC5945827; doi:10.1038/s41419-018-0627-5)
Supplement: Supplementary file 1 — Supplement Table [file 41419_2018_627_MOESM1_ESM.docx]

**Supplement Table1: The siRNA sequence of referred genes.**

| Gene Name | Sense (5’-3’) | Antisense (5’-3’) |
| --- | --- | --- |
| GAS5 siRNA1 | CUUGCCUGGACCAGCUUAAUU | UUAAGCUGGUCCAGGCAAGUU |
| GAS5 siRNA2 | CCAGCACGUUCAUACGAAUTT | AUUCGUAUGAACGUGCUGGTT |
| GAS5 siRNA3 | GCUUCCCUCUGAGUAAAUUTT | AAUUUACUCAGAGGGAAGCTT |
| CTGF siRNA1 | CCAGACCCAACUAUGAUUATT | UAAUCAUAGUUGGGUCUGGTT |
| CTGF siRNA2 | CCAAGCCUAUCAAGUUUGATT | UCAAACUUGAUAGGCUUGGTT |
| CTGF siRNA3 | GCACCAGCAUGAAGACAUATT | UAUGUCUUCAUGCUGGUGCTT |
| Negative control | UUCUCCGAACGUGUCACGUTT | ACGUGACACGUUCGGAGAATT |

**Supplement Table2: The primer of referred genes.**

| Gene Name | Forward Primer | Reverse Primer |
| --- | --- | --- |
| GAPDH | AAGGTGAAGGTCGGAGTCAA | AATGAAGGGGTCATTGATGG |
| GAS5 | AGGTATGGAGAGTCGGCTTG | GCATGCTTGCTTGTTGTGGT |
| CTGF | CAGCATGGACGTTCGTCTG | AACCACGGTTTGGTCCTTGG |
| PPAR-γ | ACCAAAGTGCAATCAAAGTGGA | ATGAGGGAGTTGGAAGGCTCT |
| C/EBP-α | TATAGGCTGGGCTTCCCCTT | AGCTTTCTGGTGTGACTCGG |
| FABP4 | ACTGGGCCAGGAATTTGACG | CTCGTGGAAGTGACGCCTT |
| miR-18a | TCGCCTAAGGTGCATCTAGTGC | CTCAACTGGTGTCGTGGAGTCGGC |
| miR-136 | TCGCCGACACTCCATTTGTTTTGAT | CTCAACTGGTGTCGTGGAGTCGGC |
| miR-26a | TCGCCGATTCAAGTAATCCAGGA | CTCAACTGGTGTCGTGGAGTCGGC |
| miR-196a | TCGCCGACTAGGTAGTTTCATGTT | CTCAACTGGTGTCGTGGAGTCGGC |
| miR-485 | TCGAAGAGGCTGGCCGTGAT | CTCAACTGGTGTCGTGGAGTCGGC |
| miR-590 | TCGCCCACGCTAATTTTATGTATAA | CTCAACTGGTGTCGTGGAGTCGGC |
| miR-876 | TCGCCGACTGGATTTCTTTGTGAA | CTCAACTGGTGTCGTGGAGTCGGC |
| miR-18a RT Primer: CTCAACTGGTGTCGTGGAGTCGGCAATTCAGTTGAGCTATCTGC | | |
| miR-136 RT Primer: CTCAACTGGTGTCGTGGAGTCGGCAATTCAGTTGAGTCCATCAT | | |
| miR-136 RT Primer: CTCAACTGGTGTCGTGGAGTCGGCAATTCAGTTGAGTCCATCAT | | |
| miR-196a RT Primer: CTCAACTGGTGTCGTGGAGTCGGCAATTCAGTTGAGCCCAACAA | | |
| miR-485 RT Primer: CTCAACTGGTGTCGTGGAGTCGGCAATTCAGTTGAGGAATTCAT | | |
| miR-590 RT Primer: CTCAACTGGTGTCGTGGAGTCGGCAATTCAGTTGAGACTAGCTT | | |
| miR-876 RT Primer: CTCAACTGGTGTCGTGGAGTCGGCAATTCAGTTGAGTGGTGATT | | |


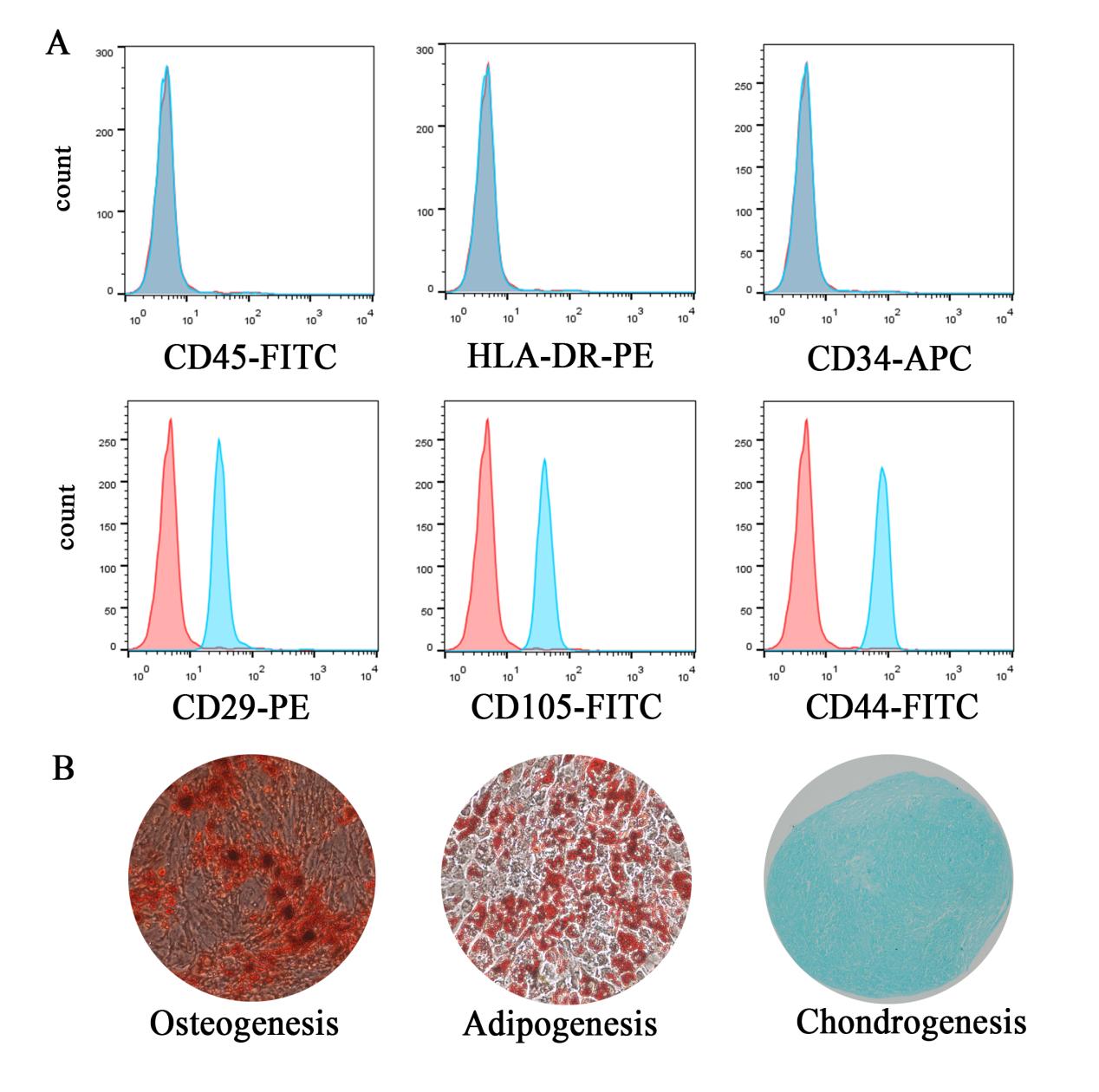


**Supplement Figure 1. Identification of human bone marrow-derived MSCs.** (A): Cell markers of MSCs were analyzed by flow cytometry, isolated cell is lack of CD45, CD34 and HLA-DR, and positive of CD29, CD105 and CD44. (B): Alizarin red S staining (×40), oil red staining (×100) and toluidine blue staining (×40) were used to detect the differentiation of MSCs.


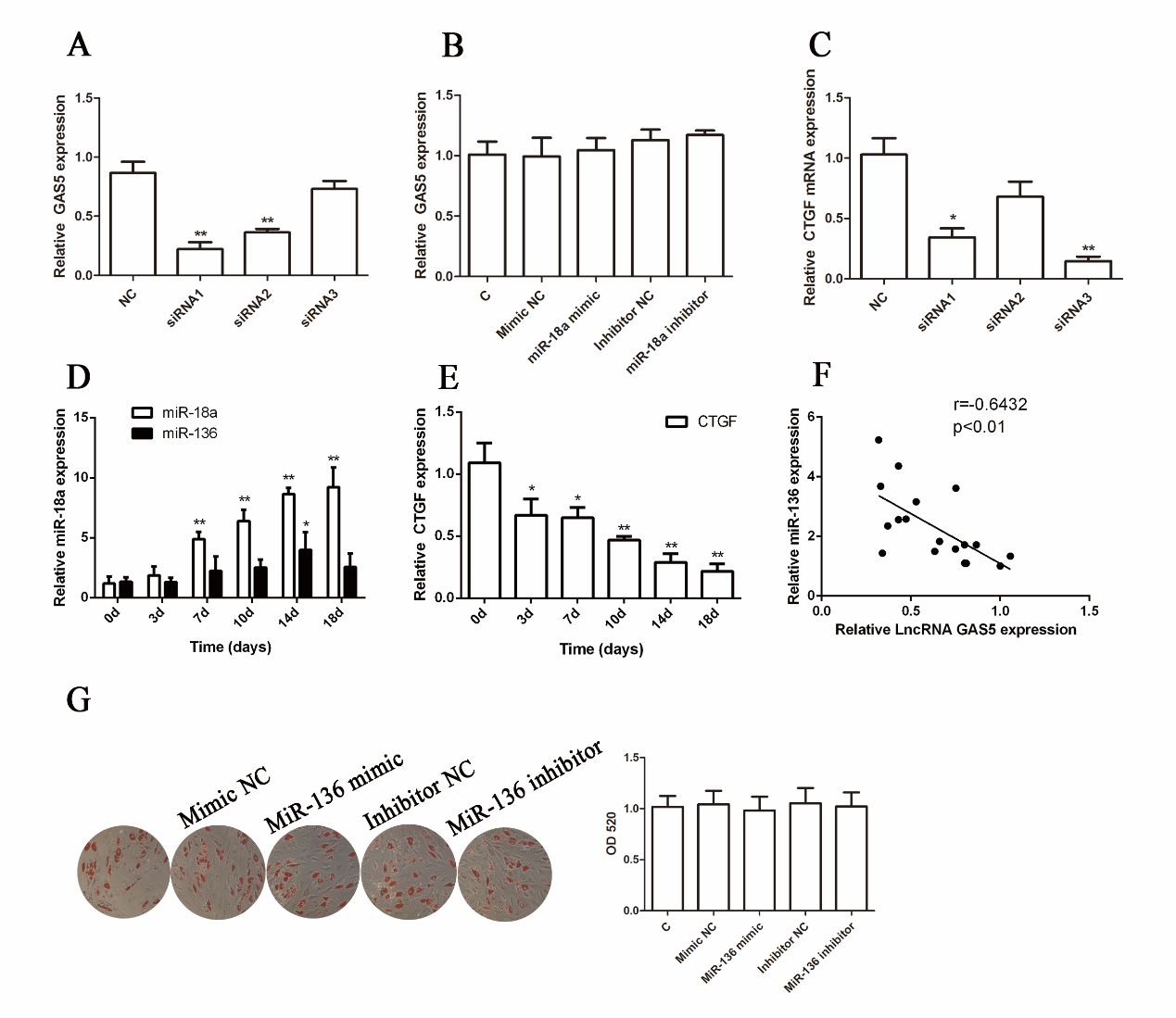


**Supplement Figure 2** (A): Knockdown efficient of GAS5 siRNAs compared with NC. (B): Relative GAS5 expression in miR-18a mimic or inhibitor transfected. (C): Knockdown efficient of CTGF siRNAs compared with NC. (D): The relative expression levels of miR-18a and miR-136 in adipogenic differentiation of MSC. (E): The relative expression levels of CTGF in adipogenic differentiation of MSC. (F): The relative expression levels of GAS5 and miR-136 in MSC adipogenic differentiation for 18 healthy donors. (G): Oil red O staining in miR-136 mimic or inhibitor transfected. Results are presented as mean ± SD (*P< 0.05, **P< 0.01).


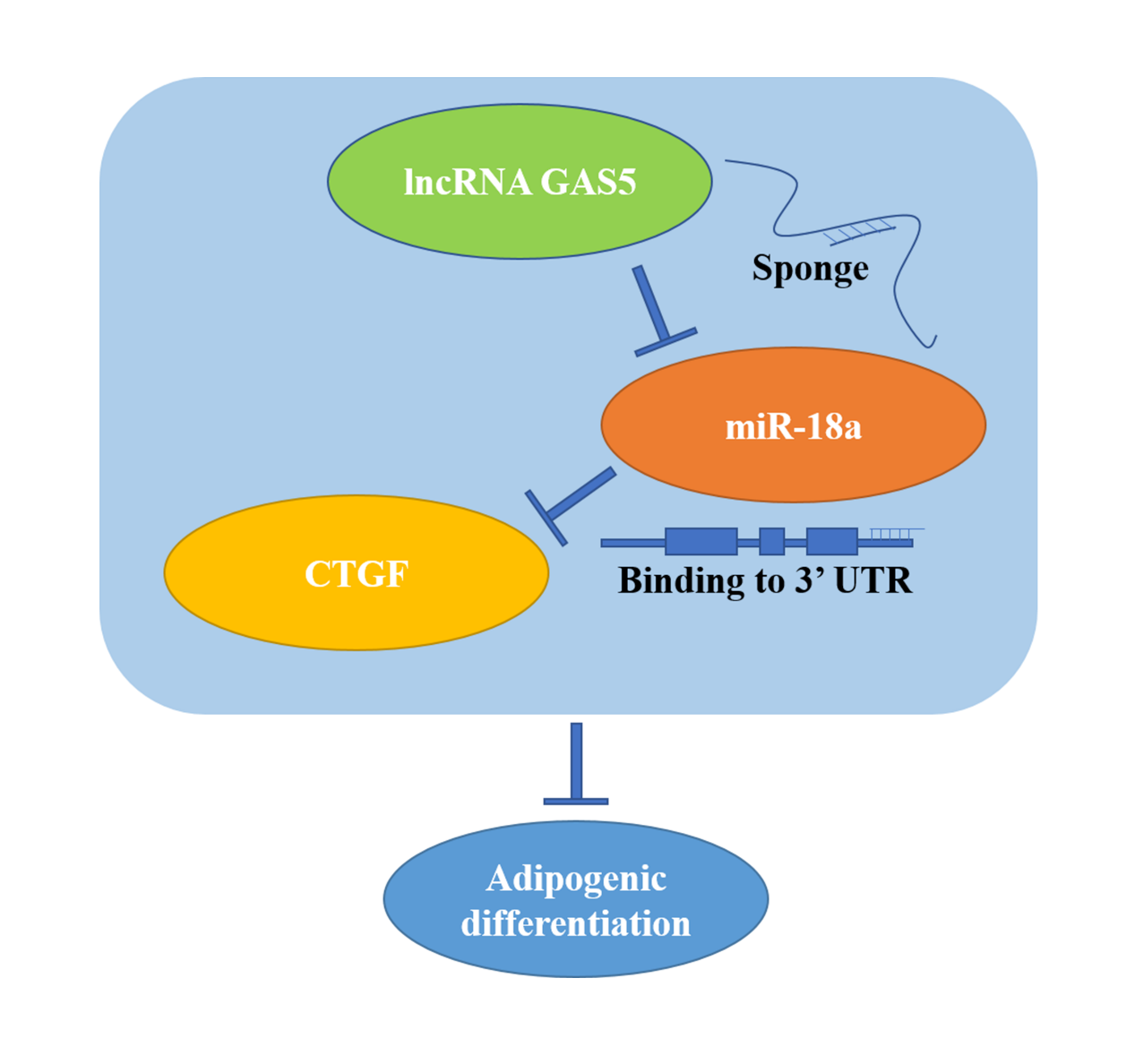


**Supplement Figure 3:** The interaction between GAS5, miR-18a and CTGF in MSC adipogenic differentiation.
