# Supplementary figures and images for: The long noncoding RNA GAS5 negatively regulates the adipogenic differentiation of MSCs by modulating the miR-18a/CTGF axis as a ceRNA
Source: Cell Death Dis. 2018 May 10;9(5):554. doi: 10.1038/s41419-018-0627-5 (PMC5945827; doi:10.1038/s41419-018-0627-5)

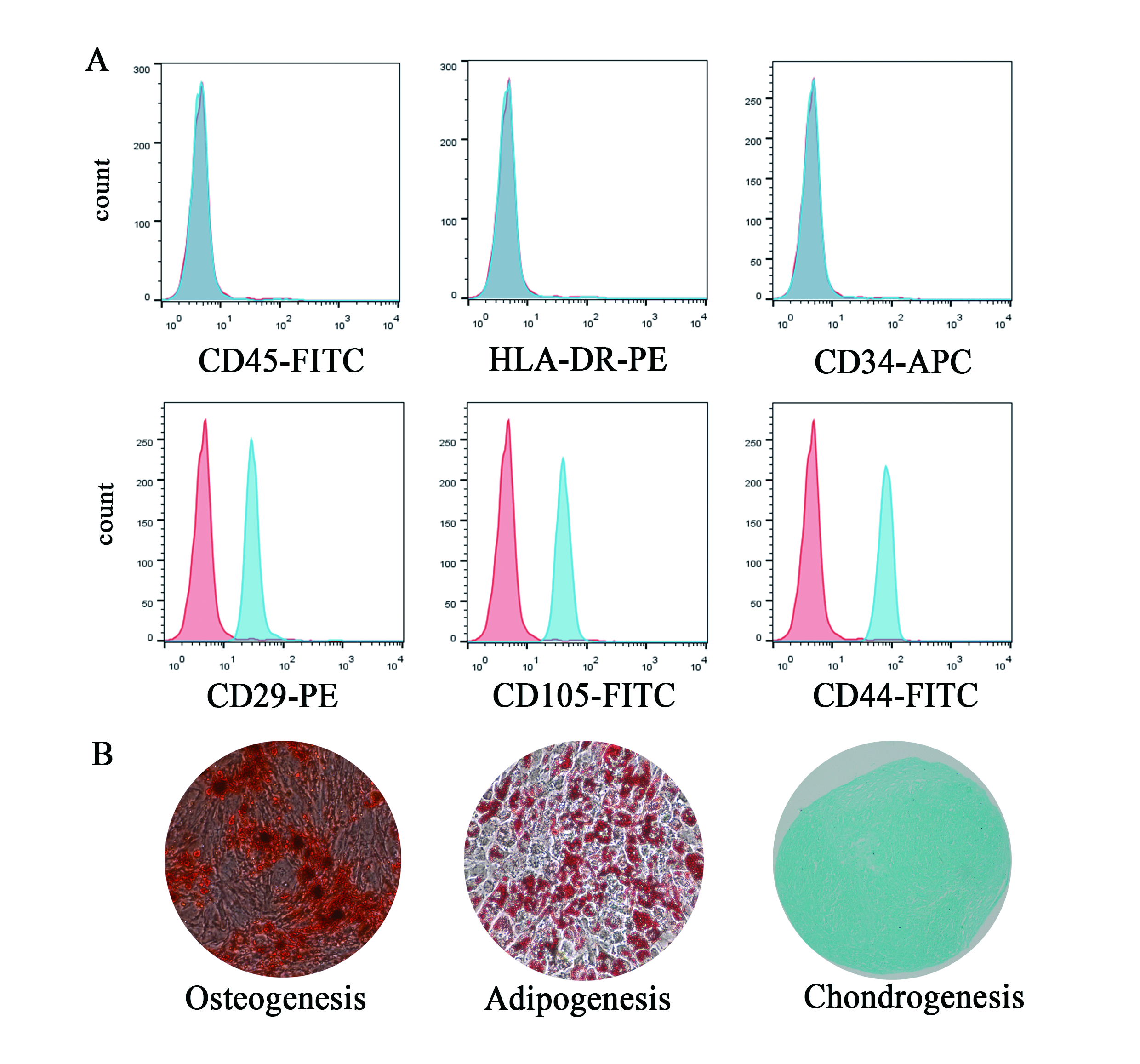

Supplement: Supplementary file 2 — Supplement Figure 1 [file 41419_2018_627_MOESM2_ESM.tif]

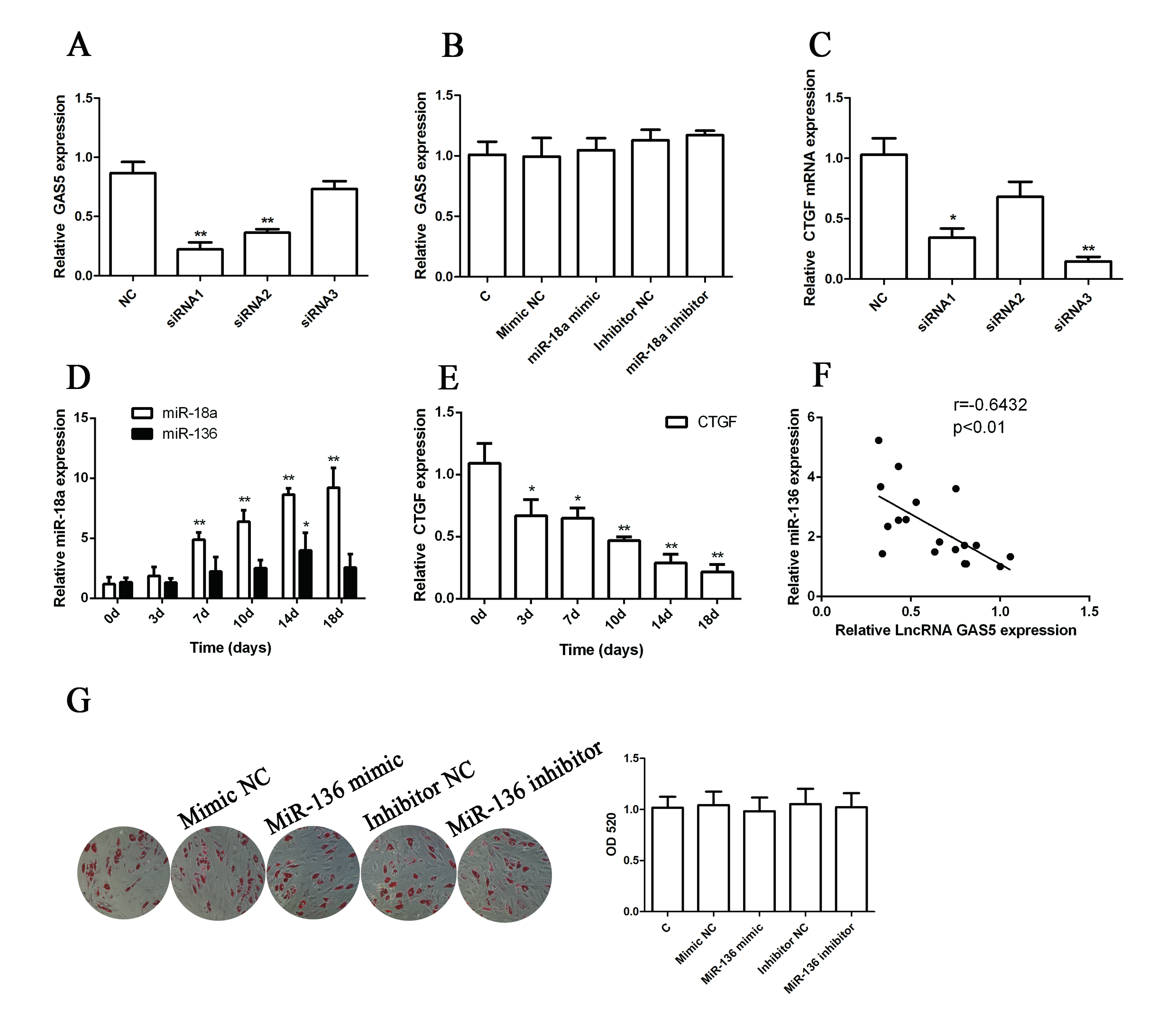

Supplement: Supplementary file 3 — Supplement Figure 2 [file 41419_2018_627_MOESM3_ESM.tif]

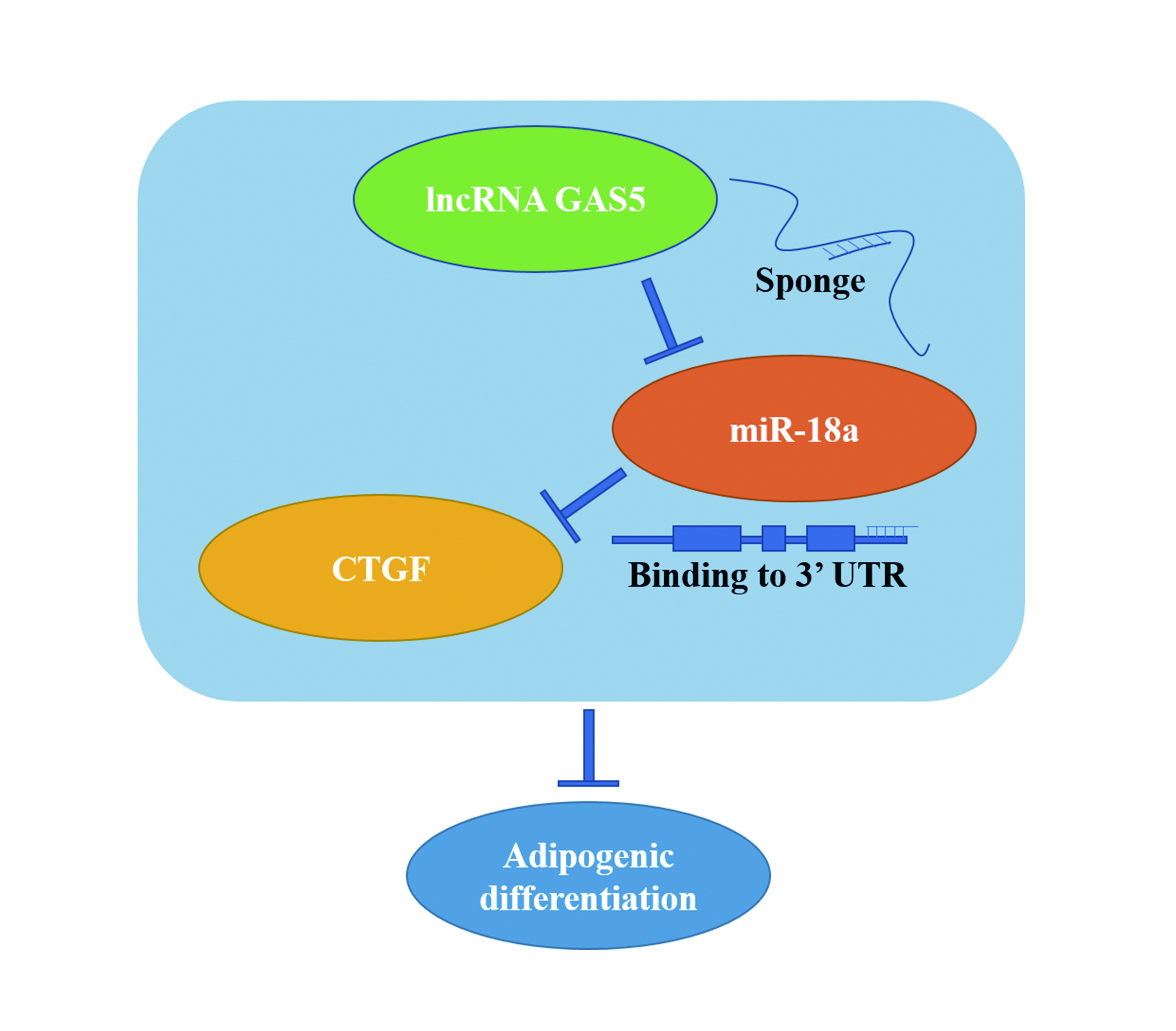

Supplement: Supplementary file 4 — Supplement Figure 3 [file 41419_2018_627_MOESM4_ESM.tif]
